# Settlement Is at the End—Common Trauma Scores Require a Critical Reassessment Due to the Possible Dynamics of Traumatic Brain Injuries in Patients’ Clinical Course

**DOI:** 10.3390/jcm13113333

**Published:** 2024-06-05

**Authors:** Jason-Alexander Hörauf, Mathias Woschek, Cora Rebecca Schindler, Rene Danilo Verboket, Thomas Lustenberger, Ingo Marzi, Philipp Störmann

**Affiliations:** 1 Department of Trauma Surgery and Orthopedics, Goethe University Frankfurt am Main, Theodor-Stern-Kai 7, 60590 Frankfurt am Main, Germany; 2 Department of Orthopedic Surgery and Traumatology, Inselspital, Freiburgstrasse 18, 3010 Bern, Switzerland

**Keywords:** polytrauma, traumatic brain injury, injury severity score, reliability, quality control

## Abstract

**Background:** Scientific studies on severely injured patients commonly utilize the Abbreviated Injury Scale (AIS) and the Injury Severity Score (ISS) for injury assessment and to characterize trauma cohorts. However, due to potential deterioration (e.g., in the case of an increasing hemorrhage) during the clinical course, the assessment of injury severity in traumatic brain injury (TBI) can be challenging. Therefore, the aim of this study was to investigate whether and to what extent the worsening of TBI affects the AIS and ISS. **Methods:** We retrospectively evaluated 80 polytrauma patients admitted to the trauma room of our level I trauma center with computed-tomography-confirmed TBI. The initial AIS, ISS, and Trauma and Injury Severity Score (TRISS) values were reevaluated after follow-up imaging. **Results:** A total of 37.5% of the patients showed a significant increase in AIS_head_ (3.7 vs. 4.1; *p* = 0.002) and the ISS (22.9 vs. 26.7, *p* = 0.0497). These changes resulted in an eight percent reduction in their TRISS-predicted survival probability (74.82% vs. 66.25%, *p* = 0.1835). **Conclusions:** The dynamic nature of intracranial hemorrhage complicates accurate injury severity assessment using the AIS and ISS, necessitating consideration in clinical studies and registries to prevent systematic bias in patient selection and subsequent data analysis.

## 1. Introduction

Traumatic brain injury (TBI) stands out as a major cause of death and long-term disability in severely injured individuals worldwide and is defined as a functional disorder or injury to the brain caused by the impact of external forces [1,2]. The severity of TBI is often classified using the Glasgow Coma Scale (GCS). A GCS score of 13–15 points is categorized as mild TBI, 9–12 as moderate TBI, and 3–8 as severe TBI [3]. Mild TBI constitutes the majority, accounting for 91% of cases, while moderate and severe TBI make up 4% and 5%, respectively [4]. Interestingly, only 39.1% of moderate or severe TBI cases result from isolated head trauma, with over 60% associated with severe multiple injuries [4].

To assess injury severity and ensure comparability for quality assurance and studies, severely injured trauma patients are typically classified using the Injury Severity Score (ISS) [5]. The ISS is calculated based on the Abbreviated Injury Scale (AIS), assigning a score between 1 (mild injury) and 6 (the most severe injury) to each injured body region on an anatomical scale [6]. After assigning the AIS values, the ISS is calculated by squaring the three highest AIS values from each of the six specified regions (AIS_A_^2^ + AIS_B_^2^ + AIS_C_^2^). The Trauma and Injury Severity Score (TRISS), developed in the 1980s, is used primarily for quality control and improving the care of severely injured patients [7,8]. Its applicability for estimating prognosis in severely injured patients has been validated in various studies [8,9,10].

The AIS and ISS are widely employed to classify injury severity in registries, clinical trials, and quality control [8,11]. In routine clinical practice, the AIS score is often estimated briefly after the initial diagnostic evaluation, potentially impacting the scoring accuracy, especially for severe TBI, which undergoes dynamic changes. For example, a femoral shaft fracture is coded once with AIS 853000.3, and no more changes are provided in the AIS regarding the injury [6]. However, this is different in craniocerebral trauma. A subdural hematoma (SDH) with a thickness of 0.5 cm on admission is assigned to AIS code 140650.3. If the SDH then increases to a thickness of 1.1 cm on control cranial computed tomography (cCT), the correct AIS code would be 140650.5 [6]. Consequently, the ISS would increase by 16 points upon recalculation, affecting the prognosis according to the TRISS.

Several studies have shown that intracranial hemorrhages can worsen during the clinical course due to a variety of different factors [12,13,14]. Since the AIS and ISS often serve as the basis for the enrollment of trauma patients in clinical retrospective and prospective studies, the adjustment of the AIS and ISS in the context of time-dependent deterioration in TBI is crucial for precise classification. However, to our knowledge, no study has yet investigated the impact of TBI progression on injury severity as assessed by the AIS and ISS. We hypothesize that a significant proportion of polytrauma patients with traumatic brain injury will exhibit injury progression, resulting in increased AIS and ISS scores. Therefore, this retrospective study aimed to analyze cases requiring adjustments of the AIS, ISS, and TRISS due to dynamic changes in TBI, assessing the extent of change in these cases.

## 2. Materials and Methods

The retrospective data access for this study was carried out between 1 January 2021 and 31 July 2021 and included all patients admitted to the trauma room of our level 1 trauma center between 1 April 2019 and 30 November 2020 who had an AIS_Head_ value of ≥2 at initial clinical radiological examination and were subsequently treated in the intensive care unit (ICU). The initial AIS and ISS scores obtained after trauma room care were retrieved from the hospital information system (HIS). The patient’s inpatient course was then analyzed, and the AIS, ISS, and TRISS values were recalculated after clinical radiological follow-up to reflect possible dynamic changes. As an in-house standard, in close consultation with the neurosurgery department, CT examination of the head is typically repeated 4–6 h after the initial detection of intracranial pathology. In the clinical course, if there is a lack of a proper awakening response, magnetic resonance imaging (MRI) is performed as the standard procedure, with no specified timing for this.

According to Boyd et al., we calculated the TRISS. This method is based on the ISS, the Revised Trauma Score (RTS, including GCS score, systolic blood pressure, and respiratory rate) [15], the patient’s age, and the type of injury (blunt or penetrating trauma) [16].

Patients younger than 18 years of age were excluded, as were cases lacking information regarding the size/extent of the intracranial bleeding or cases without a follow-up imaging assessment. In addition to potential quantitative changes in the aforementioned scores, the reasons for AIS changes and the time in which the changes occurred were also determined. This retrospective study was approved by the local ethics committee of the medical faculty of the Johann Wolfgang Goethe University of Frankfurt am Main (No.: 19-491; date of approval 11 December 2019) and follows the Strengthening The Reporting of Observational Studies in Epidemiology (STROBE) and Reporting of studies Conducted using Observational Routinely-collected Data (RECORD) guidelines for observational studies [17,18]. Informed consent was waived given the retrospective nature of the study. All retrospective data were obtained from the HIS. To protect the information and confidentiality of the patients, the names of the patients were not documented. An anonymous study ID was assigned to each patient on a data collection sheet. The patient’s study ID was kept on a separate record sheet and only accessible to the corresponding author and the research coordinator. The Mann–Whitney U test was used to compare the mean values of the AIS, ISS, and TRISS. The Chi-square test was used to assess the association between categorial variables. A two-tailed *p*-value < 0.05 was considered statistically significant. The descriptive and comparative statistical analyses were performed using GraphPad Prism 10 for Mac (Dotmatics, San Diego, CA, USA). Values are reported as mean ± standard deviation (SD) for continuous variables and as percentages for categorical variables.

## 3. Results

### 3.1. Demographic Data

A total of 80 patients were enrolled in the study. Their mean age was 54.6 years (±20.9 SD). Among the patients, 61.3% were male. On admission, the mean GCS score was 7.9 points (±5.1 SD). All patients were treated in the ICU, and the average length of stay in the ICU was 8.6 days (±7.4 SD). The initial mean ISS was 22.9 points (±14.7 SD), with 57.5% (*n* = 46) having an ISS ≥ 16 points. The mortality rate was 11.3% (*n* = 9), and the median survival time of these patients after admission was 1.9 days (±2 SD).

### 3.2. Mechanism of Injury

Falls with an assumed fall height of less than three meters constituted the primary cause of the accidents, accounting for 31.3% (*n* = 25), followed by traffic accidents (22.5%, *n* = 18) and bicycle falls (17.5%, *n* = 14). Further details on the trauma mechanisms are provided in Table 1.

### 3.3. Classification of Injury Severity According to the Glasgow Coma Scale

The mean initial GCS score in the emergency room was 7.98 points (±5.1 SD). Following the current recommendations for the classification of TBI using the GCS, severe TBI was present in 52.5% of cases (*n* = 42), moderate TBI in 31.25% (*n* = 25) of the cases, and mild TBI in 16.25% (*n* = 13).

### 3.4. AIS and ISS Changes

**Figure 1:**
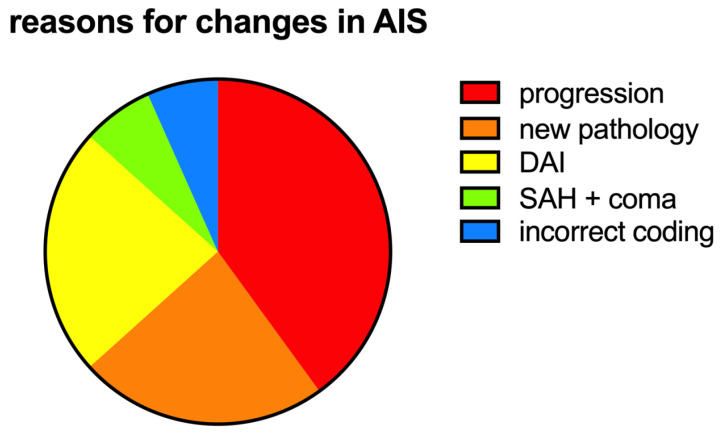
Reasons for changes in the initial AIS. The majority of reasons for altering the Abbreviated Injury Scale (AIS) scores were represented by an increase in existing hemorrhage (40%, *n* = 12), the appearance of new hemorrhage pathology (23%, *n* = 7), and MR-based detection of diffuse axonal injury (23%, *n* = 7) on follow-up imaging. In 7% (*n* = 2) of cases, subarachnoid hematoma with intraventricular involvement was detected on admission, which, according to the AIS, was classified as more severe in the further clinical course if unconsciousness persisted for more than 6 h. In 7% (*n* = 2), reevaluation of initial CT scan revealed an incorrect (too low) AIS score on admission because the bleeding area was measured as too small.

In total, retrospective correction of the AIS or ISS values was conducted in 30 patients (37.5%). The primary reason for adjusting the AIS/ISS values was a change in or renewal of the findings in the CT and MR follow-up imaging (*n* = 26, 86.7%). Among the observed changes, 40% (*n* = 12) were due to a significant enlargement of the index pathology. In 23.3% (*n* = 7) of cases, the change resulted from a new pathology in terms of an additional bleeding entity. Moreover, in 23.3% (*n* = 7) of cases, the adjustment of the AIS value was due to MR-based detection of diffuse axonal injury (DAI). Detailed information on the reasons for the AIS changes is provided in Figure 1.

On average, AIS adjustment occurred approximately 6.8 h after admission (±5.7 h SD, min: 3 h, max: 26 h) when based on changes detected by CT. When based on changes identified by MR, AIS adjustment occurred, on average, approximately 147 h later (±122 h SD, min: 12 h, max: 312 h).

**Figure 2:**
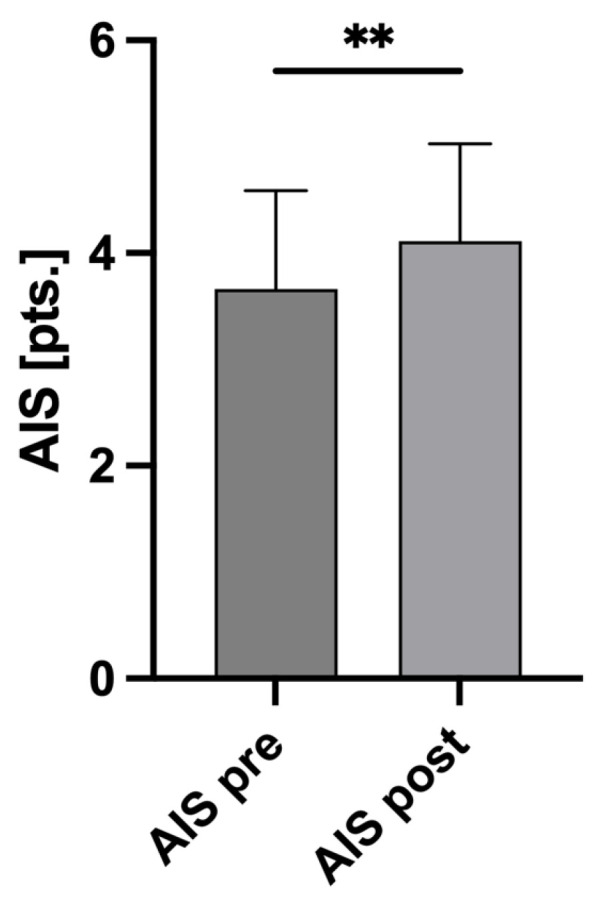
Changes in AIS score. Before adjustment, the average Abbreviated Injury Scale (AIS) score for the head of all 80 patients included was 3.66 points (pts.) ± 0.93 SD (AIS pre). After adjustment, the AIS score was significantly higher at 4.11 pts. ± 0.91 SD (AIS post), *p* = 0.002 **. Mann–Whitney U test was used to compare the mean AIS values.

**Figure 3:**
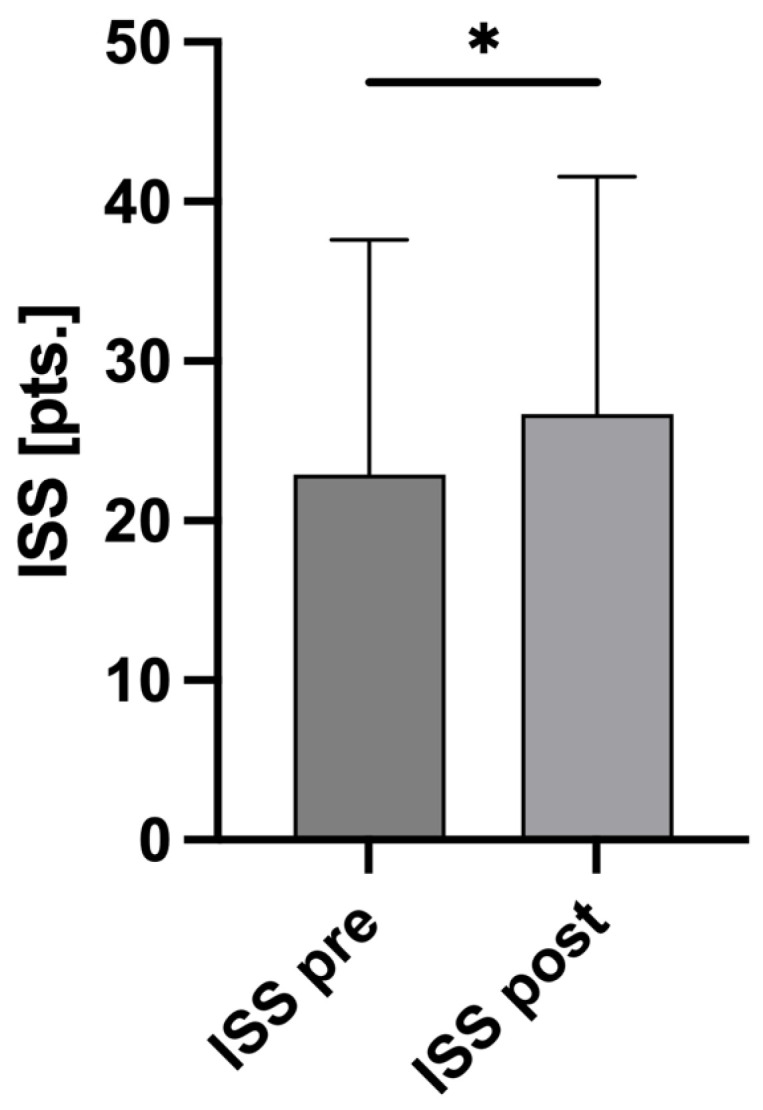
Changes in ISS. Based on the changes to the Abbreviated Injury Scale (AIS) score for the head, a recalculation of the Injury Severity Score (ISS) was performed. The average ISS of all 80 included patients at admission was 22.89 points (pts.) ± 14.73 SD (ISS pre). After adjustment of AIS scores, the average ISS increased significantly to 26.68 pts. ± 14.89 SD (ISS post), *p* = 0.0497 *. Mann–Whitney U test was used to compare the mean ISS values.

As a result, the AIS scores after reevaluation were significantly higher than at admission (AIS_pre_ 3.66 ± 0.93 SD vs. AIS_post_ 4.11 ± 0.91 SD; *p* = 0.0020; Figure 2). Based on these AIS changes, the ISSs were recalculated, also showing a significant increase (ISS_pre_ 22.89 ± 14.73 SD vs. ISS_post_ 26.68 ± 14.89 SD; *p* = 0.0497; Figure 3).

### 3.5. Influence of the Altered AIS Scores on the TRISS

**Figure 4:**
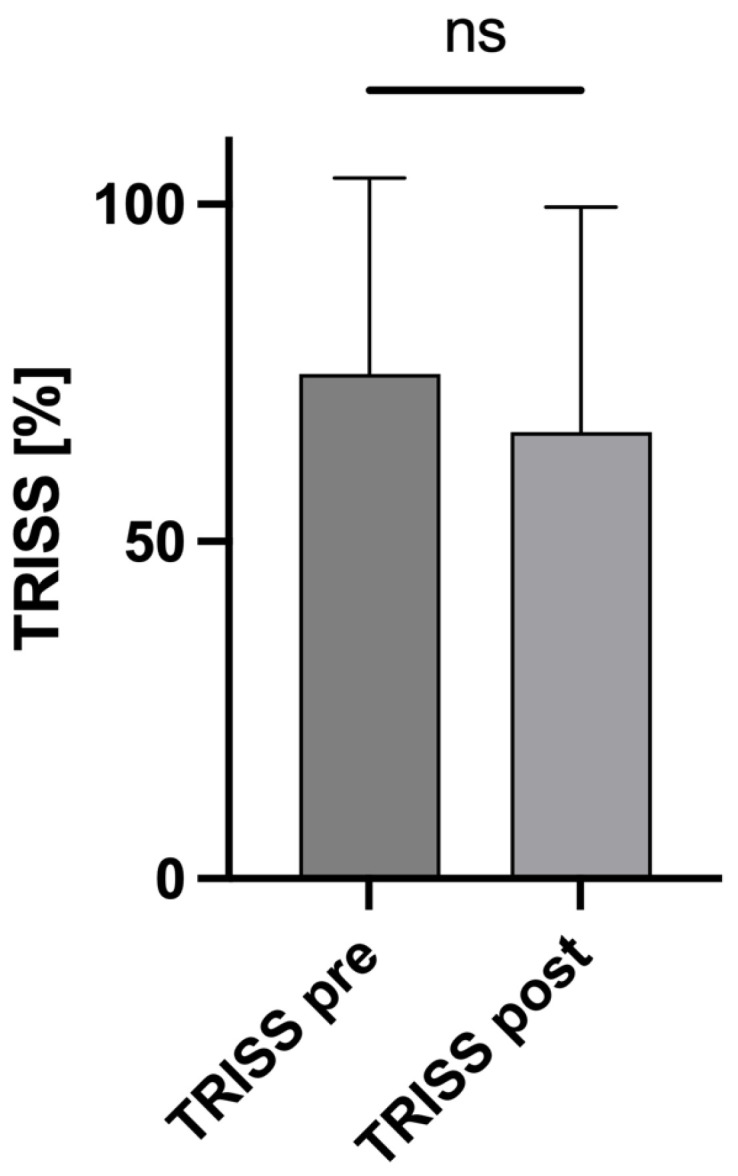
Changes in the TRISS. Based on the Abbreviated Injury Scale (AIS) scores for the head at the time of admission and the resulting Injury Severity Scores (ISSs), the average Trauma Score and Injury Severity Score (TRISS) was 74.82% ± 29.07 SD (TRISS pre). After correction for AIS and ISS values, TRISS decreased to 66.25% ± 33.3 SD (TRISS post), *p* = 0.1835. Mann–Whitney U test was used to compare the mean TRISS values. ns: not significant.

To assess the impact of AIS alteration on the survival probability of the trauma patients, a post hoc analysis of the TRISS was performed. Therefore, the TRISS values were calculated in a subgroup analysis of patients with relevant changes in their AIS scores. Based on AIS_head_ at the time of admission and the resulting ISS, the average TRISS was 74.82% (±29.07 SD). After correction for the AIS and ISS values, the average TRISS decreased to 66.25% (±33.3 SD; *p* = 0.1835), resulting in an average reduction in the survival probability of 8% (Figure 4).

### 3.6. Influence of the Altered AIS/ISS Values on Clinical Outcome

To examine the potential impact of changes in the AIS/ISS scores on clinical outcomes, we compared the AIS-corrected group (*n* = 30) to the non-corrected group (*n* = 50). The overall mortality rate in this study was 11.3%. The subgroup analysis revealed that only 1 of the 30 patients (3.33%) in the AIS-corrected group died, compared to 8 of the 50 patients (16.0%) in the non-corrected group (*p* = 0.1425). There were no significant differences in the initial GCS scores (AIS-corrected: 8.0 points ± 4.9 SD vs. non-corrected AIS score: 7.9 ± 5.3 SD; *p* = 0.8534) or in age (AIS-corrected: 53.6 years ± 20.92 SD vs. non-corrected AIS score: 55.2 years ± 21.01 SD; *p* = 0.7276) between the two groups. However, the deceased were significantly older (74.1 years ± 21.51 SD) than the survivors (52.2 years ± 19.58 SD, *p* = 0.0035). Patients in the AIS-corrected group had significantly longer ICU stays (12.2 ± 8.9 days) compared to those in the non-corrected group (6.5 days ± 5.4 SD; *p* = 0.0009).

## 4. Discussion

In this retrospective study involving 80 patients with TBI, we demonstrated that in 37.5% of cases, the initial severity of head injury assessed using the AIS worsened during the clinical course. Based on our data, the AIS and ISS values calculated after the completion of emergency room diagnostics are subject to high variability and must be reevaluated and adjusted according to new findings during follow-up.

Since the 1980s, scoring systems for severely injured patients have been used and developed to classify injury severity and measure treatment success and specific patient outcomes [19]. Moreover, scores such as the ISS are used to define and compare cohorts for clinical trials and registries [11]. These scoring systems have been repeatedly revised and adjusted, particularly for prognosis assessment and local and international quality control regarding preventable death in trauma [8,20]. The weaknesses of the existing scoring systems are primarily the under-triage of trauma severity and the lack of consideration of patient-specific factors (e.g., preexisting medical conditions, long-term medication, age, gender, etc.) [21]. Newer scoring systems such as the Revised Injury Severity Score II (RISC II) and the Sequential Trauma Score (STS) show better discrimination of these factors and allow for a better estimation of prognosis, but they require a larger number of factors and data [20,22]. Even though the ISS represents only a part of these modern scores, a falsely low ISS may lead to inaccuracy and thus potentially the exclusion of patients in TBI studies and general systematic bias in clinical trials, as shown in our study.

The clinical outcome after TBI is determined primarily by the primary injury caused directly by mechanical forces during the initial insult and secondarily by subsequent secondary injury, which leads to further tissue and cellular damage through biochemical, cellular, and (patho-)physiological mechanisms [23]. As an anatomical score, the AIS score can depict primary damage but is insufficient for assessing secondary damage.

Regarding TBI in patients with multiple injuries, it has been shown that the initial GCS value correlates only to a small extent with the later clinical outcome of patients [24]. Factors that may influence the reliability and validity of the GCS include intoxication, sedation, hemodynamic instability in the sense of shock, and intubation [25]. In these cases, clinical assessment of patients is based particularly on their radiographic injury sequelae on initial imaging. Therefore, the CT-based Marshall criteria [26] and later the Rotterdam criteria [27] were developed to predict early mortality after moderate and severe TBI. Nevertheless, with the exception of the size of the bleeding and intrusion into the ventricular system in subarachnoid hemorrhages in the initial assessment of the AIS, these criteria do not impact the currently used trauma scores. This issue has been indirectly the subject of several studies aimed at improving the classification of TBI and providing a more accurate prognosis for patient outcomes [10,28]. However, a major problem in TBI research is that, on the one hand, neurosurgeons and neurologists commonly choose the GCS and the Marshall and Rotterdam grading systems, respectively, as the inclusion criteria for studies, while, on the other hand, trauma surgeons frequently use the AIS or ISS values as inclusion criteria for investigations and studies on TBIs [29]. Moreover, observational studies and studies on biomarkers often require an ISS ≥ 16 upon admission to define multiple trauma [30,31]. Another major problem is that the patient population included in these trials is often inhomogeneous despite having clear inclusion criteria (e.g., GCS score < 9). Thus, Bendinelli et al. revealed that a group with GCS scores of 3–5 when compared with a group with GCS scores of 6–8 had more frequent episodes of hypoxia, more often required a craniectomy, and had higher mortality. The authors emphasize that patients with signs of head injury and a prehospital GCS score below 9 appear to be far too heterogeneous to be similarly treated, benchmarked, and similarly included in clinical trials [32].

In a retrospective multicenter study, Bossers et al. demonstrated that approximately 13% of head-injured patients with apparently mild TBIs, defined by an initial GCS score of 13–15 at presentation, were at substantial risk of being more severely injured than initially classified [33]. This is consistent with the results of our study, which demonstrated such an effect in 37.5% of patients. The higher percentage of the discrepancy between the initial assessment and post hoc determination of injury severity in the present study may be explained by the fact that patients with more severe TBIs on average, and in particular with severe concomitant injuries in the sense of polytrauma, were included in our study.

Given the potential dynamics of intracranial hemorrhage, especially in the first hours after trauma, immediate injury severity classification based on the AIS is a notable problem. We showed that even in short-term cCT follow-up, despite an initially rather small intracranial hemorrhage, a relevant enlargement of the initial hemorrhage often occurs, and thus the ISS increases significantly. In the context of retrospective studies, this can still be taken into account during later evaluations. However, in prospective studies with clear inclusion criteria and study protocols or in biobanks already started in the trauma bay, retrospective enrollment is impossible and may result in suitable study patients being excluded or incorrectly assigned to a comparison cohort due to underestimated injury severity.

In the present study, reassessment of the AIS and ISS scores resulted in an increase in the ISS ≥ 25 in 15 patients (50%). This, in turn, would have led to the enrollment of these patients in the so-called national “Network Trauma Research” (Netzwerk Traumaforschung, NTF) biobank for fluid samples of polytraumatized patients, which was initiated in 2013 by the NTF task force of the German Trauma Society (Deutsche Gesellschaft für Unfallchirurgie, DGU) [34]. The data presented here suggest that polytrauma patients with relevant TBIs (e.g., AIS score ≥ 3) should be included in studies as a precaution when it is estimated that the TBI may progress to avoid losing valuable blood samples. In the further course, the ISS can be reevaluated after imaging controls, and in the case of ISS instability, the sample material can be discarded again.

Both the present results and literature review show that the clinical presentation of an isolated TBI but also that of a TBI in a polytraumatized patient can deteriorate rapidly. Thus, the injury severity of this patient collective must not be underestimated under any circumstances. The German Society for Neurosurgery (DGNC) recommends in its S2e guideline “traumatic brain injury in adults” performing a cCT control immediately in case of neurological deterioration or in case of a lack of recovery or unconscious patients after 4–8 h after the initial imaging [35]. The level 3 guideline on the treatment of patients with severe/multiple injuries of the DGU further recommends a follow-up CT within 8 h if any signs of injury were seen on the initial head CT [36]. In our department, the standard procedure for any evidence of intracranial hemorrhage is to perform a follow-up CT scan at 4–6 h.

Since the outcome of polytraumatized patients is significantly influenced by the severity of the TBI, it is essential to include this factor in the decision-making process, especially as part of the initial care concept. Unfortunately, there are currently no standardized guidelines for the acute treatment of patients with severe TBI and polytrauma, leading to global variations in clinical practices [37]. The 2019 World Society of Emergency Surgery (WSES) consensus conference proposed guidelines for managing adult patients with TBI and polytrauma within the first 24 h. The guidelines recommended urgent neurosurgical intervention for salvageable patients with life-threatening brain lesions after achieving bleeding control for a critical hemorrhage [38]. However, no specific recommendations were provided for subsequently managing the extracranial injuries. A commonly practiced principle of acute care for polytrauma patients in many hospitals is “Damage Control Surgery” (DCS) [39] and delayed final fracture fixation to minimize “secondary hits” to the injured brain [40,41]. Although these orthopedic practices are believed to reduce the likelihood of exacerbating brain injury outcomes by minimizing hemodynamic complications and mitigating increased inflammatory responses, there is an ongoing debate on whether these trauma practices indeed improve the outcomes for TBI patients and whether the impact of these “secondary hits” genuinely influences the pathobiology of such injuries [42,43].

All in all, for severe TBI in polytraumatized patients, the injury severity, especially to the head, should not be underestimated due to potential dynamic changes. Even if neurosurgical intervention is not initially necessary, patients should be closely monitored, and early CT follow-up is crucial, particularly in the acute phase after trauma with evidence of intracranial bleeding. Given the potential risk of further (iatrogenic) worsening of a TBI during the surgical treatment of polytraumatized patients, the option of DCS should be considered in the initial decision-making process to minimize secondary hits in the acute inflammatory phase. Furthermore, the postoperative logistic processes should be adapted. Thus, during initial surgical treatment, subsequent follow-up imaging could be implemented between the surgical and intensive care phases to avoid unnecessary transport times and conserve limited personnel resources.

## 5. Limitations

Our study has several limitations. The restricted number of included patients and the retrospective, monocentric study design represent notable constraints. Due to the retrospective nature of this study, there are no fixed time points for controls or follow-up imaging, with the examination modalities being based primarily on the patient’s clinical course. Nonetheless, this study’s substantial percentage of significant radiological changes underscores the importance of rigorous clinical and radiological follow-up in TBIs. Further prospective studies are essential to conclusively validate the observed effect. A promising avenue in this regard is the TBI module already established in a pilot phase by the professional societies DGU and DGNC as an extension of the existing TraumaRegister DGU^®^ (TR-DGU) [44].

## 6. Conclusions

Due to the significant dynamic changes in the initial radiographic findings in TBIs, which persist at high percentages despite modern intensive care therapy concepts, achieving accurate trauma injury scoring through conventional methods is challenging and remains a major obstacle. Common trauma scores may exhibit substantial differences in injury severity depending on the time of assessment, thereby significantly influencing their prognostic accuracy. However, determining an optimal time for score calculation in the clinical course is challenging due to the distinct requirements of these scores. Hence, the present study underscores the importance of a critical evaluation of the scores in trauma patients with TBI, taking into account possible dynamic changes to attain the most accurate results. Since the AIS and ISS scores frequently serve as the criteria for enrolling trauma patients in clinical studies, it is essential to anticipate potential changes in the injury severity to prevent systematic bias in patient selection and subsequent study analyses.

## Figures and Tables

**Table 1:** Demographic and clinical characteristics.

	*n* = 80 Patients
age (years) ± SD	54.6 ± 20.9
sex (male)	49 (61.3%)
GCS_admission_ (pts.) ± SD	7.9 ± 5.1
ICU_stay_ (days) ± SD	8.6 ± 7.4
AIS_pre_ (pts.) ± SD	3.66 ± 0.93
AIS_post_ (pts.) ± SD	4.11 ± 0.91
ISS_pre_ (pts.) ± SD	22.89 ± 14.73
ISS_post_ (pts.) ± SD	26.68 ± 14.89
TRISS_pre_ (%) ± SD	74.82 ± 29.07
TRISS_post_ (%) ± SD	66.25 ± 33.3
mortality (*n*)	9 (11.3%)
Injury mechanism (*n*)	
fall below 3 m	25 (31.3%)
traffic accident	18 (22.5%)
bicycle fall	14 (17.5%)
fall over 3 m	14 (17.5%)
assault	8 (10%)

Shown are demographic and clinical characteristics as well as the injury mechanisms of TBI patients that led to presentation to the emergency department. In one case, the mechanism was not documented. Abbreviations: AIS: Abbreviated Injury Scale; GCS: Glasgow Coma Scale; ICU: intensive care unit; ISS: Injury Severity Score; SD: standard deviation; pts.: points; TRISS: Trauma Score and Injury Severity Score.

## Data Availability

The data are contained within the article.
